# Surface-Functionalization of PP7 Virus-Like Particles with Spytag for Bioconjugation Applications

**DOI:** 10.1007/s12033-026-01554-5

**Published:** 2026-01-27

**Authors:** Milad Kheirvari, Ebenezer Tumban

**Affiliations:** School of Veterinary Medicine, Graduate Program in One Health Sciences, Texas Tech University, Amarillo, TX 79106 USA

**Keywords:** Virus-like particles, PP7, Qβ, Spytag/Spycatcher, Antigen conjugation, Nanocarriers, Vaccine platforms, Molecular engineering

## Abstract

Virus-like particles (VLPs) are protein-based nanoscale assemblies derived from structural proteins of viruses. They are non-infectious scaffolds and are highly immunogenic; thus, they widely used as scaffolds to display and enhance the immunogenicity of less immunogenic foreign antigens. Different approaches, including genetic insertions, can be used to display foreign antigens on VLPs; however, some of these approaches have limitations, i.e., inability of viral coat proteins to tolerate a foreign insertion. In this study, we assessed the tolerability of bacteriophage Qβ and PP7 coat proteins to insertions of Spytag003 or Spytag peptides; we assessed whether the insertions of these peptides to the coat proteins will affect the ability of the recombinant coat proteins to assemble into VLPs; chimeric VLPs displaying these peptides can be used to conjugate antigens for vaccine studies. While the insertion of Spytag003 peptide at either the *N* or *C* termini of the coat protein of Qβ and at the *C* terminus of the coat protein of PP7 did not affect the expression of the recombinant proteins, the expressed proteins were not soluble. In contrast, insertion of the shorter Spytag at either the *N* terminus or AB-loop of PP7 gave rise to soluble proteins that assembled into VLPs. PP7-Spytag VLPs were successfully conjugated with SpyCatcher003 and a fungal antigen. Immunization studies revealed that Spycatcher003 protein conjugated on PP7-SpyTag VLPs elicited significantly higher anti-Spycatcher003 antibody titers compared to unconjugated Spycatcher003 (*p* = 0.0286). Together, these findings establish proof of concept that PP7-Spytag VLPs should be explored as a platform to conjugate foreign proteins.

## Introduction

Peptide and protein antigens are alternatives to traditional vaccines such as live-attenuated vaccines and inactivated vaccines; they exhibit excellent safety profiles [1–4]. Peptides and some protein vaccines are however less immunogenic and require conjugation or display on larger immunogens such as Keyhole limpet hemocyanin to enhance their immunogenicity [5, 6]. Within the last decade, virus-like particles (VLPs) have gained attention as a display platform for peptide and some protein antigens [7, 8]. VLPs are self-assembling structural proteins (cage-like structures derived from viruses) that look like viruses except that they lack viral genome; they are highly immunogenic [7, 9, 10]. Given their immunogenic properties, VLPs have been used as display platforms for less immunogenic antigens [7, 8]; less immunogenic antigens are displayed multivalently on the surface of VLPs. Peptides or protein antigens can be displayed on VLPs by genetic insertion or by chemical conjugation. The Chemical conjugation approach is expensive and complicated while the genetic insertion approach is cheap and can be scaled up for large-scale production. However, the genetic insertion approach can be very challenging with some antigens. Genetic insertion of some antigens on the coat proteins can sometimes prevent the coat proteins from assembling to form VLPs [11, 12]. Recently, a new bio-conjugation system that allows for the conjugation of a protein on VLPs was developed [13–16].

This bio-conjugation system consists of a 13 amino acid peptide (Spytag; AHIVMVDAYKPTK) and its binding partner, 138 amino acids protein (Spycatcher). Spytag is genetically inserted on the VLPs while Spycatcher is expressed as a recombinant protein linked to an antigen of interest. Mixing of the recombinant VLPs (displaying Spytag) with a Spycatcher linked to a protein of interest leads to the conjugation of the protein on the VLPs. This approach has been used to bio-conjugate foreign antigens on the surfaces of VLPs derived from bacteriophage AP205, norovirus, hepatitis B, porcine circovirus type 2, etc. [13–18]. However, the original SpyTag/SpyCatcher system was derived from *Streptococcus pyogenes*, and therefore antibodies against SpyCatcher are frequently found in humans due to natural exposure to this bacterium. This high level of pre-existing immunity limits its usefulness for vaccine applications [19] and can attenuate the efficacity of the system and consequently any antigen conjugated using this system. To obviate this limitation, a new bio-conjugation system (Spytag003/Spycatcher003) was recently developed [20]. Spytag003 (**RGVP**HIVMVDAYK**RY**K) is three amino acids longer than of prototype Spytag (AHIVMVDAYKPTK) and differs from prototype Spytag by 6 amino acids (highlighted in purple bold text). Spycatcher003, the binding partner of Spytag003, differs from the prototype Spycatcher by 13 amino acids [20]. Unlike the prototype Spytag/Spycatcher system, Spytag003/spycatcher003 reaction is 400 times faster and has less pre-existing antibodies in the population [20]. This new bio-conjugation system has never been explored to display an antigen on any VLP platform. In this study, we explored for the first time the potential of genetically inserting Spytag003 on the coat proteins of two bacteriophages, PP7 and Q*β*. Specifically, we assessed whether the insertion of Spytag003 (or Spytag) on the coat proteins will prevent the coat proteins from assembling into chimeric VLPs. We chose coat proteins from PP7 and Qβ bacteriophages because their VLPs have not be used to bio-conjugate foreign antigens using any of the Spytag/Spycatcher conjugation systems, thus offering an opportunity to expand the diversity of VLP platforms available for antigen display.

## Materials and Methods

### Insertions of Spytag003 or Spytag into the Coat Proteins of Qβ and PP7 and Assessment for Solubility

**Fig. 1:**
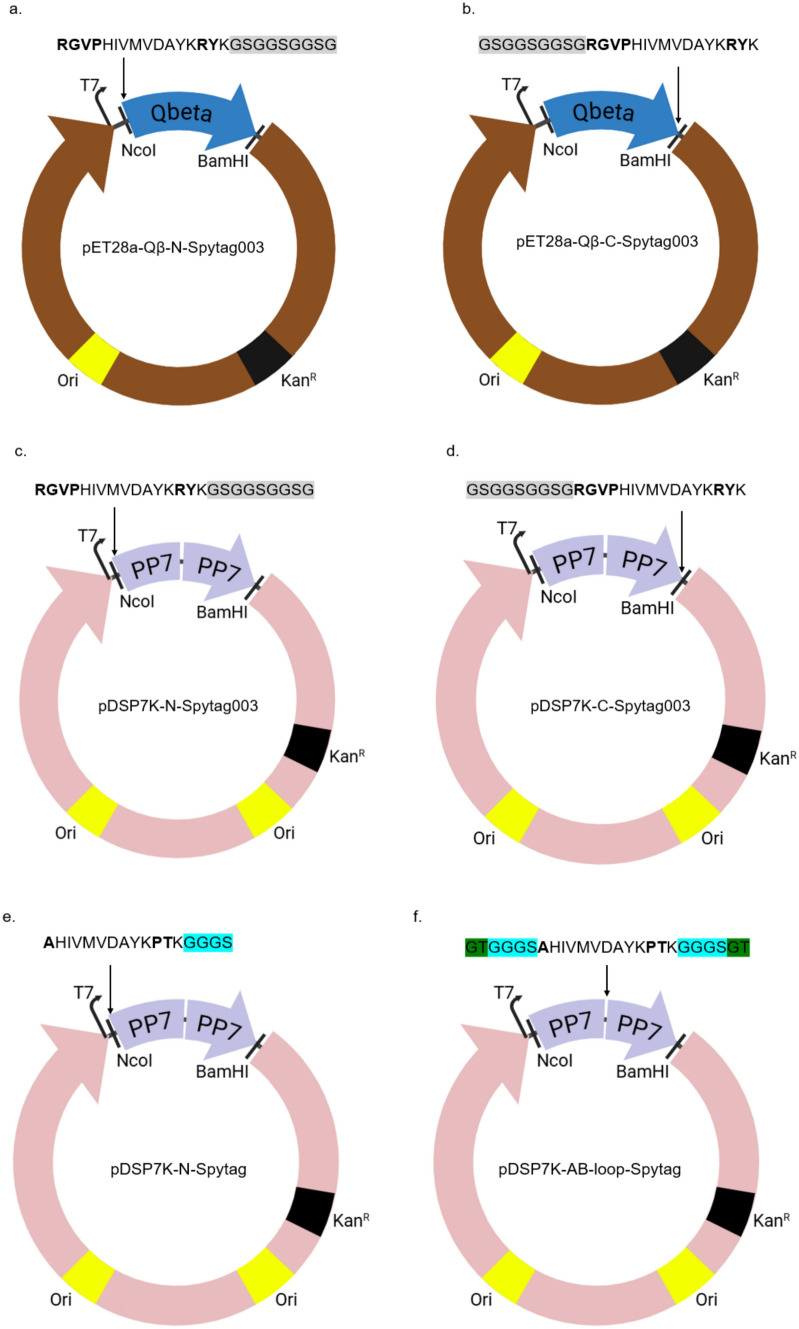
Design of Qβ-Spytag003, PP7-Spytag003, and PP7-Spytag expression constructs. Schematics of **a** pET28a–Qβ-N-Spytag003, **b** pET28a–Qβ-C-Spytag003, **c** pDSP7K–PP7-N-Spytag003, and **d** pDSP7K–PP7-C-Spytag003 vectors showing the insertions of Spytag003 peptide (**RGVP**HIVMVDAYK**RY**K) at the *N* terminus or *C* termini of the coat proteins of Qβ and single-chain dimer PP7 (pDSP7k). Linker sequence, GSGGSGGSG, is highlighted in gray background. Insertions were done using NcoI and BamHI restriction sites. Schematics of **e** pDSP7K–PP7-N-Spytag and **f** pDSP7K–PP7-Spytag AB-loop vectors showing the insertions of Spytag peptide (**A**HIVMVDAYK**PT**K) in the *N* terminus and in the AB-loop (in-between amino acids 138 and 139) of the coat protein of the single-chain dimer of PP7. Linker sequences (GGGS) used for insertion are highlighted in turquoise background. For AB-loop insertion, a KpnI site (in green background) flanking the linker sequences was included for cloning. The six amino acids that makes Spytag003 different from Spytag are highlighted in bold text in panels a–d, while the three amino acids that makes Spytag different from Spytag003 are highlighted in bold text in panels e and f

The DNA sequence encoding Spytag003 peptide (RGVPHIVMVDAYKRYK) was inserted at either the *N* terminus or the *C* terminus of the coat proteins of bacteriophage Qβ (GenBank: ACY07236.1); the peptide was also inserted to the coat proteins of the single-chain dimer of bacteriophage PP7 [21] (Fig. 1A–D). A nine-amino acid flexible linker (GSGGSGGSG) was incorporated in-between each of the Spytag003 sequence and Qβ and PP7 coat proteins to promote flexibility of the tag on the coat proteins and to reduce steric hindrance. The coat protein sequences with the insertions were codon-optimized for *E. coli* expression and were synthesized and cloned into pET28a expression vector by Epoch Life Sciences. In addition to the insertions of Spytag003 peptide to the coat protein of PP7, the prototype Spytag (AHIVMVDAYKPTK) was separately inserted at either the *N* terminus or the AB-loop of the coat protein (Fig. 1E and F). For *N*-terminal insertion, a four-amino acid flexible linker (GGGS) was included in-between Spytag and the coat protein; for AB-loop insertion, Spytag (flanked by the linker and KpnI site on both sides) was inserted between amino acids 138 and 139. For PP7, the insertion was done in pDSP7K expression vector, which expresses the single-chain dimer of PP7 coat protein [21].

For protein expression, the plasmids were separately transformed into *E. coli* C41(DE3), BL21 Star pLysS, or Rosetta pLysS. Single colonies were inoculated into Luria–Bertani (LB) broth and grown at 37 °C until an OD_600_ of ~ 0.6 was reached. Protein expression was induced with 0.5 mM isopropyl β-D-1-thiogalactopyranoside (IPTG) at 37 °C for 4 h [22]. Cells were harvested by centrifugation (5000 × g, 10 min, 4 °C), lysed and lysates were run on SDS-PAGE. Bacterial cells that showed the highest level of protein expression were screened for solubility as follows: bacterial pellets expressing PP7 and Qβ coat proteins with Spytag003 or Spytag insertions were lysed with BugBuster® (Millipore Sigma; Cat 70,584-M) [23] supplemented with 0.1% Triton-X 100, and soluble (supernatant) and insoluble (pellet dissolved in 8 M urea) fractions were analyzed by SDS-PAGE.

Insoluble bacterial pellets expressing recombinant coat proteins of PP7 and Qβ were subjected to sequential lysis as follows. Cells were first lysed with Sepharose column buffer (SCB; 20 mM Tris–HCl pH 7.4, 100 mM NaCl, 0.1 mM MgSO_4_), and supernatants were collected. The remaining pellets were subsequently re-lysed with Borax buffer (10 mM sodium tetraborate decahydrate, and 10 mM NaCl), followed by BugBuster reagent. Residual insoluble material was then solubilized using increasing concentrations of urea (0.5, 1, and 8 M, sequentially). Supernatants from each step were analyzed for the presence of target proteins by SDS-PAGE.

### Purification of Recombinant Coat Proteins and Assessment of Assembly into Chimeric VLPs

Recombinant coat proteins were purified from large-scale culture by lysing bacterial pellet with BugBuster protein extraction reagent. 0.1% triton X-100 was added to bacterial lysates, the lysates were centrifuged at 10,000 rpm for 10 min and supernatant were run on 23, 29, and 35% Optiprep gradient (Millipore Sigma; Cat D1556) [24]. The gradient was run at 50,300 rpm at 16 °C for 3.5 h. For the purification of PP7 coat proteins with Spytag insertions, bacterial pellets were lysed with 0.2% lysozyme solution supplemented 0.05% with deoxycholate. DNase I (0.01 mg/mL) and MgCl₂ (0.2 M) were added to lysates and the mixtures were incubated at 37 °C for 1 h. Lysates were clarified by centrifugation at 3,700 rpm for 30 min at 4 °C, and ammonium sulfate (70% saturation) was added to the supernatant. Samples were incubated on ice for 30 min and centrifuged at 10,000 rpm for 10 min at 4 °C. Pellets were resuspended in SCB buffer (20 mM Tris–HCl, 100 mM NaCl, 0.1 mM MgSO_4_, pH 7.4) and clarified by centrifugation (10,000 rpm, 10 min, 4 °C). Following ammonium sulfate precipitation, supernatants were loaded onto a Sepharose CL-4B size exclusion column equilibrated with SCB buffer (20 mM Tris–HCl, 100 mM NaCl, 0.1 mM MgSO_4_, pH 7.4) and purified as previously described [25]. Briefly, the samples were allowed to flow into the beads by gravity. Once all the samples were in the beads, SCB buffer was connected to the column and fractions (3 mL) from the column were collected and analyzed on SDS-PAGE gel.

### Expression and Purification of Spycatcher003-Ag2/PRA-CSA Recombinant Protein

Ag2/PRA antigen (amino acids 1–106, which has been shown to offer similar levels of protection compared to the full-length sequence [26]) was fused to CSA antigen; both antigens are from *C. posadasii*. A three-amino acid linker peptide (GGG) was included between the two proteins to increase their flexibility. The recombinant gene (Ag2/PRA-CSA) was fused to the *C* terminus of Spycatcher003 gene, to enable bio-conjugation on VLPs. Six-histidine residues were added to the *N* terminus of Spycatcher003 gene to enable purification of the fusion protein by affinity chromatography. The gene fragment was codon optimized for bacterial infection and was synthesized and cloned into pET28a by Epoch Life Sciences. Protein expression was induced at OD_600_ of 0.6 with 0.5 mM IPTG for 3 h. For purification, bacterial pellet was first lysed with 0.2% lysozyme solution followed by lysis with 8 M urea buffer (20 mM NaH_2_PO_4_, 20 mM Na_2_HPO_4_, supplemented with 400 mM NaCl, 50 mM imidazole, 10% tween 20, 10 mM beta-mercaptoethanol, pH 7.5). The mixture was sonicated, spun, and the supernatant added to Ni–NTA beads (Qiagen; Cat 30,210). The beads were washed 5X with 8 M urea buffer. Recombinant protein was eluted from the column using elution buffer (8 M urea, 20 mM NaH_2_PO4, 20 mM Na_2_HPO4, 300 mM NaCl, 250 mM imidazole, pH 7.5). Eluted protein was dialyzed and refolded by buffer exchange with refolding buffer 1 (0.5 M urea, 20 mM NaH_2_PO_4_, 5 mM reduced glutathione, 0.5 mM oxidized glutathione, 0.5 M arginine, 300 mM NaCl, 10% glycerol, pH 7.5) followed by another buffer exchange with refolding buffer 2 (20 mM NaH_2_PO4, 150 mM NaCl, 10% glycerol, pH 7.5).

### Conjugation of Spycatcher003 and Spycatcher003-Ag2/PRA-CSA to PP7-N-Spytag VLPs

For conjugation, PP7-N-Spytag VLPs were incubated with Spycatcher003 (Bio-Rad; Cat TZC025) at 1:3.25 molar ratio, respectively. To conjugate Spycatcher003-Ag2/PRA-CSA to the VLPs, purified Spycatcher003-Ag2/PRA-CSA was conjugated to PP7-N-Spytag VLPs at 1:0.43 molar ratio. All reactions were carried out at room temperature for 3 h. Conjugation products were analyzed by SDS-PAGE, and band intensities were compared using ImageJ software to assess relative conjugation efficiency.

### Transmission Electron Microscopy (TEM) Analysis

Purified VLPs were absorbed onto carbon-coated, glow-discharged copper grids for 2 min. Grids were washed with distilled water and negatively stained with 2% uranyl acetate for 2 min. VLPs were visualized using a Hitachi H-7500 transmission electron microscope at 50,000X–70,000X.

### Immunization of Mice

Because these are pilot studies, groups of four Balb/c mice were immunized subcutaneously with PP7-N-Spytag VLPs conjugated with Spycatcher003 (1.5 μg of Spycatcher003 and 1 μg PP7-N-Spytag VLPs mixture). Control mice were immunized with 1.5 μg of SpyCatcher003 or 1 μg of unconjugated PP7-N-Spytag VLPs. All immunizations were administered twice without adjuvant at two-week intervals. Two weeks after the final immunization, whole blood was collected, and sera were prepared for antibody analysis.

### Enzyme-Linked Immunosorbent Assays (ELISA)

Antibodies in sera were determined by ELISA as follows: High-binding 96-well plates were coated overnight at 4 °C with 500 ng of Spycatcher003. Plates were washed three times with PBS + 0.05% Tween-20 (PBST) and blocked with 5% non-fat dry milk in PBST for 1 h at room temperature. Serial dilutions of mouse sera in blocking buffer were added and incubated for 2 h at room temperature. Plates were washed, and IgG (total IgG) was detected with horseradish peroxidase (HRP)-conjugated goat anti-mouse IgG, followed by TMB substrate. Reactions were stopped with 1 M HCl, and absorbance was measured at 450 nm using a microplate reader. Endpoint titers were calculated as the highest serum dilution giving an optical density above background.

### Statistical Analysis

Statistical analysis was performed using GraphPad Prism software. Comparisons between groups were made using Mann–Whitney *U* tests. *P* values < 0.05 were considered statistically significant.

## Results

### The Coat Proteins of Qβ and PP7 with Spytag003 Insertions Were Successfully Expressed But Did Not Assemble into VLPs

**Fig. 2:**
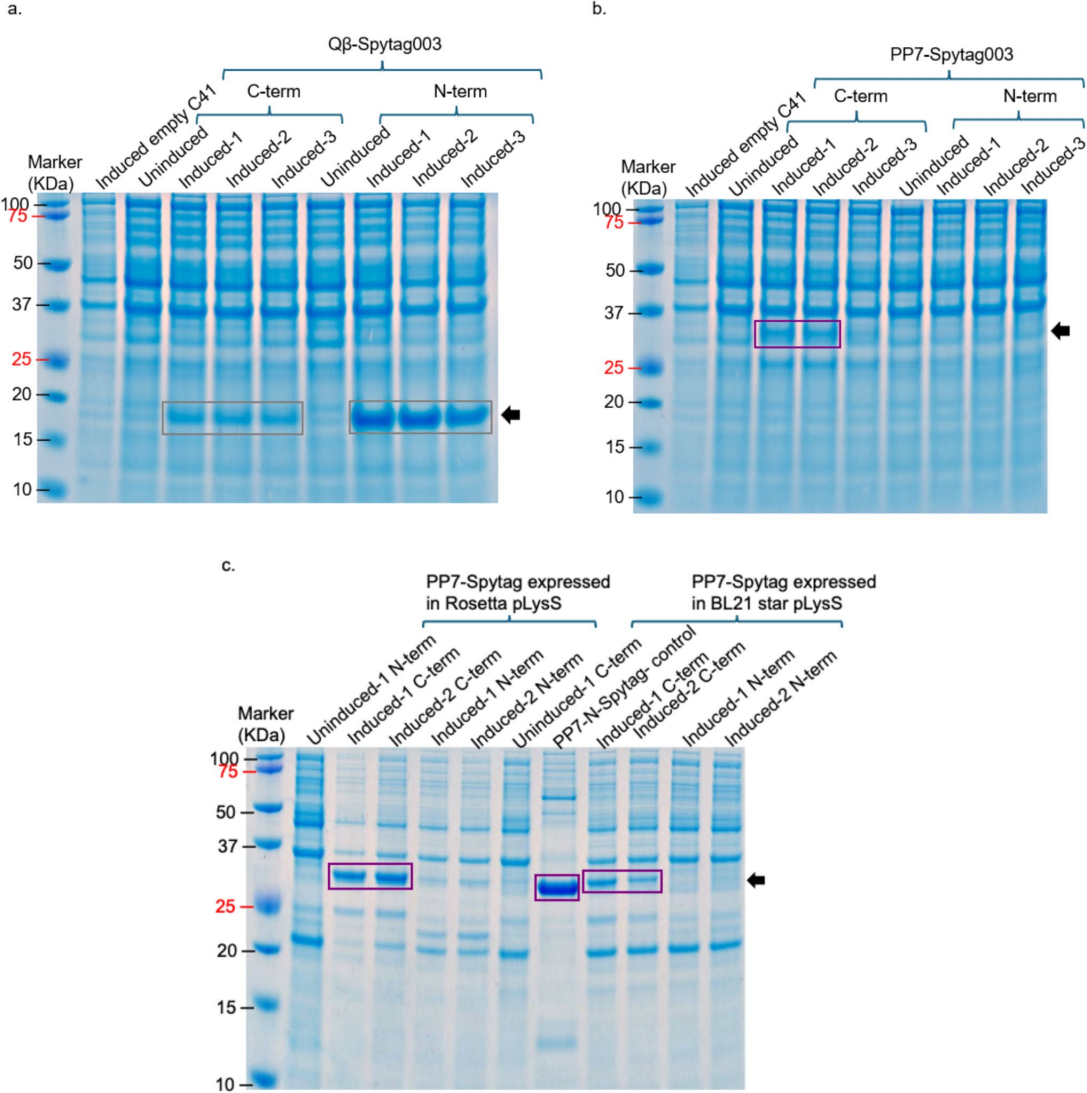
SDS-PAGE of small-scale expression of the recombinant coat proteins of Qβ-Spytag003 and PP7-Spytag003 in C41 (DE3), Rosetta (DE3) pLysS, and BL21 Star (DE3) pLysS. **a** Expression of Qβ-N-Spytag003 and Qβ-C-Spytag003 coat proteins in C41 cells. **b** Expression of PP7-N-SpyTag003 and PP7-C-Spytag003 coat proteins in C41. **c** Expression of PP7-N-Spytag003 and PP7-C-Spytag003 coat proteins in Rosetta cells and BL21 Star cells. Four hours after induction, bacteria were pelleted, lysed with 8 M urea and lysates were run on SDS-PAGE gels. In all cases, recombinant coat protein was induced with 0.5 mM IPTG at 37 °C for 4 h. Induced-1, Induced-2, and Induced-3 represent independent bacterial colonies collected from the agar plate transformed with the plasmids expressing the coat proteins above. Arrows indicate expected proteins sizes (~ 16.78 KDa for Qβ-N-Spytag003 and ~ 30.59 KDa for PP7-C-Spytag003)

To explore the feasibility of genetically inserting Spytag003 into the coat proteins of Qβ and PP7, four constructs were designed with Spytag003 inserted at either the *N* terminus or *C* terminus of the coat proteins of Qβ and PP7 (single-chain dimer): pET28a-Qβ-N-Spytag003, pET28a-Qβ-C-Spytag003, pDSP7K-N-Spytag003, and pDSP7K-C-Spytag003 (Fig. 1). The coat proteins of Qβ with *N*- and *C*-terminal insertions (Qβ-N-Spytag003 and Qβ-C-Spytag003) and the coat protein of PP7 with *N*- and *C*-terminal insertions (PP7-N-Spytag003 and PP7-C-Spytag003) were successfully expressed in C41 cells. As shown in Fig. 2A and B, bands of ~ 16.78 KDa and ~ 30.59 KDa (which correspond to expected sizes of the coat proteins of Qβ and PP7 with insertions, respectively) were observed in the induced samples but not in uninduced samples. The expression levels of Qβ with Spytag003 inserted at the *N* terminus was better than the insertion at the *C* terminus. PP7 with the insertion of Spytag003 at the *N* terminus of the coat protein did not show any signs of protein expression. To evaluate whether lack of visible levels of protein expression was due to the host cell in question, we transformed pDSP7K-N-Spytag (the expression vector that contained the protein) into other strains of *E. coli* (Rosetta pLysS and BL21 Star pLysS); Rosetta 2 is a strain of BL21 cells with additional copies of genes that code for rare tRNA; this promotes the expression of heterologous proteins with rare codons in bacteria [27]. BL21 Star, on the other hand, is a strain of BL21 cells with a mutation in rne131 gene which enhances RNA stability and consequently high levels of protein expression. As shown in Fig. 2C, only PP7 with the insertion of Spytag003 at the *C* terminus was expressed in both cells.

**Fig. 3:**
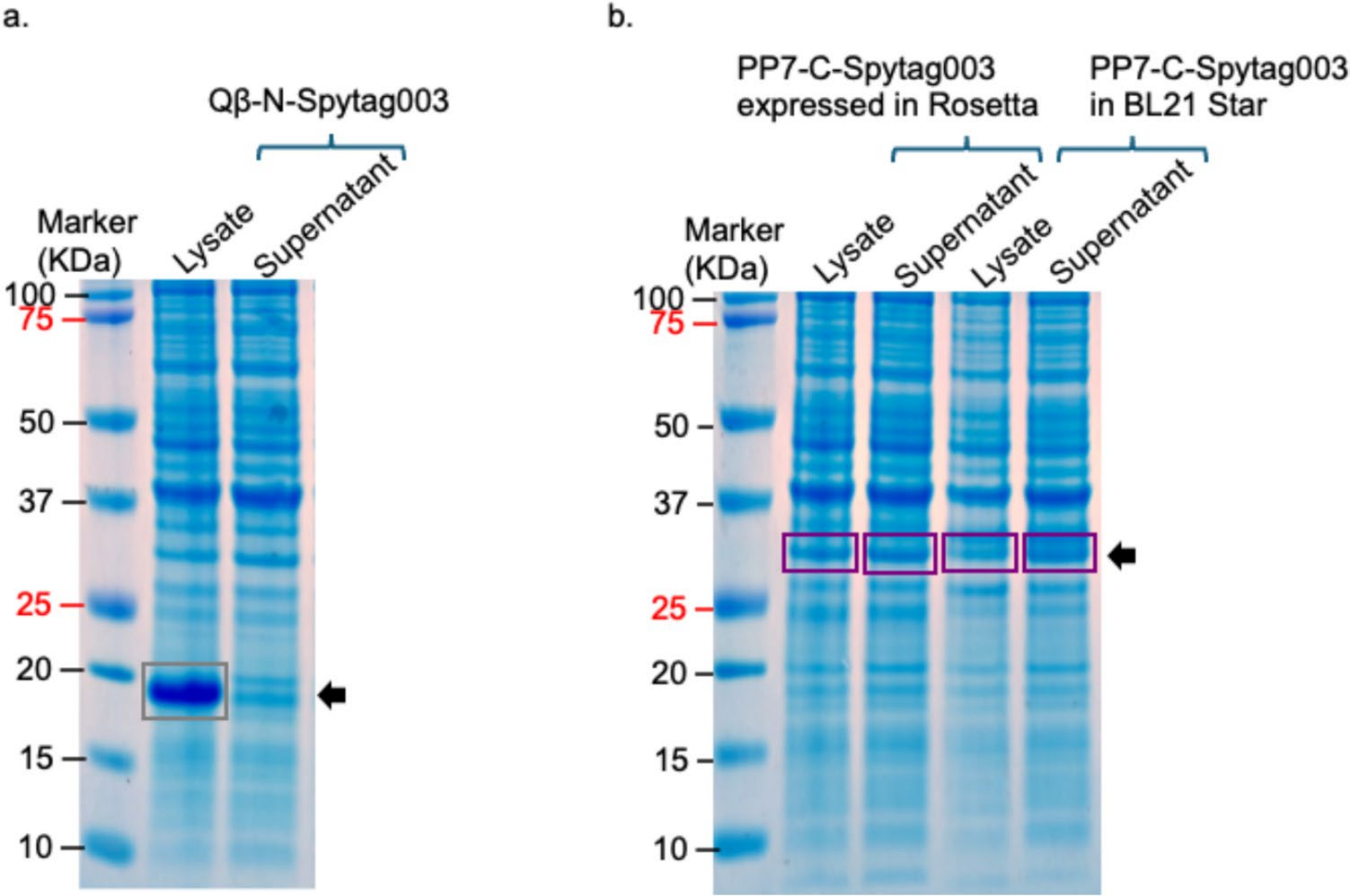
Solubility assessment of the coat proteins of Qβ-Spytag003 coat protein and PP7-Spytag003 coat protein. **a** C41 bacterial pellet expressing Qβ-N-Spytag003 coat protein was lysed with BugBuster reagent supplemented with 0.1% triton-X-100, centrifuged and the supernatant and lysate analyzed on SDS-PAGE gel. **b** Bacterial pellet expressing PP7-C-Spytag003 coat protein was lysed as in panel (a) and supernatant & lysates analyzed on SDS-PAGE gel. Arrows indicate expected proteins sizes (~ 16.78 KDa for Qβ-N-Spytag003 and ~ 30.59 KDa for PP7-C-Spytag003)

To determine if expressed proteins were soluble for purification purposes and for the purpose of evaluating assembly to VLPs, we conducted solubility studies. Bacterial pellets expressing Qβ-N-Spytag003, Qβ-C-Spytag003, and PP7-C-Spytag003 coat proteins were lysed with BugBuster reagent supplemented with 0.2% Triton-X 100. Only Qβ-N-Spytag003 and PP7-C-Spytag003 were partially soluble after lysis with the reagent (Fig. 3).

**Fig. 4:**
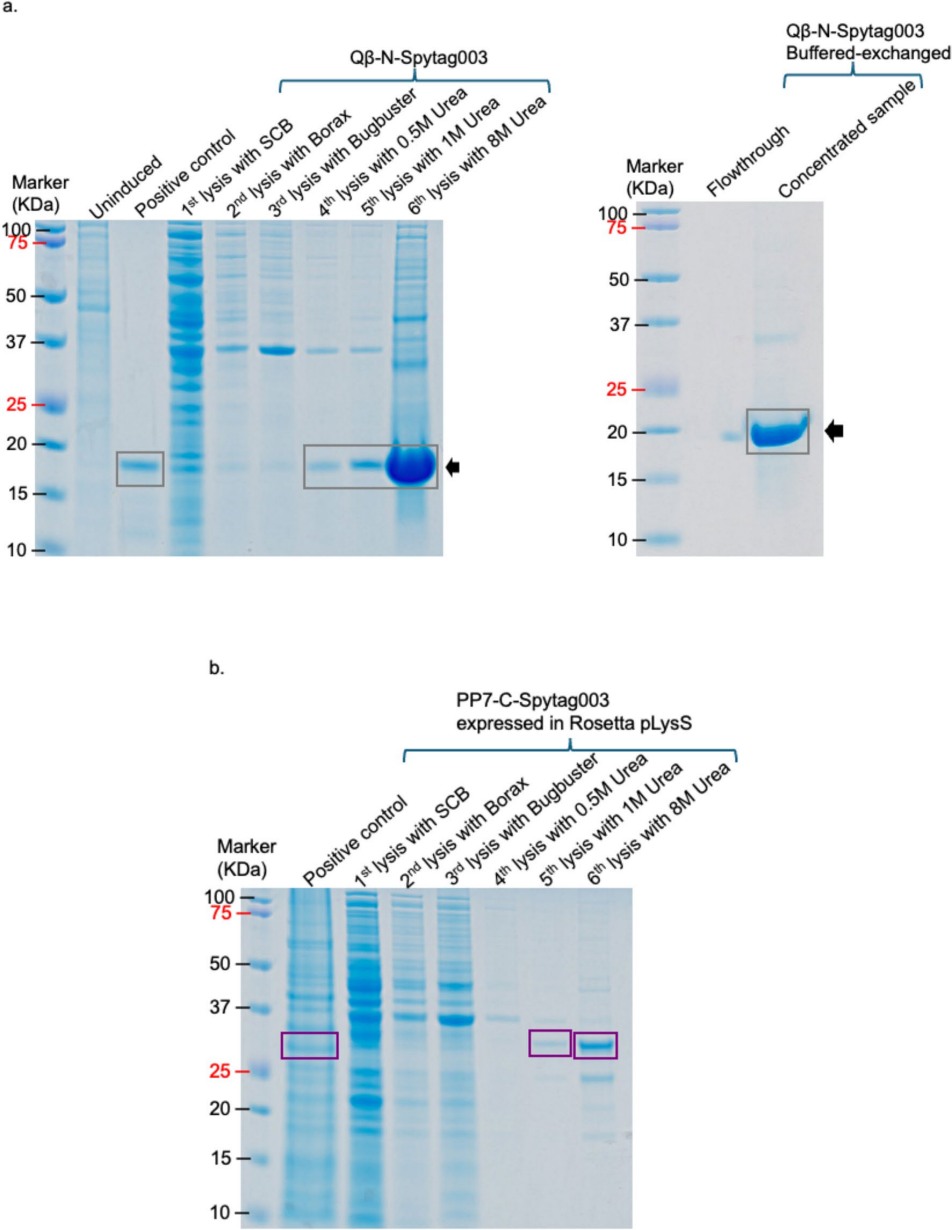

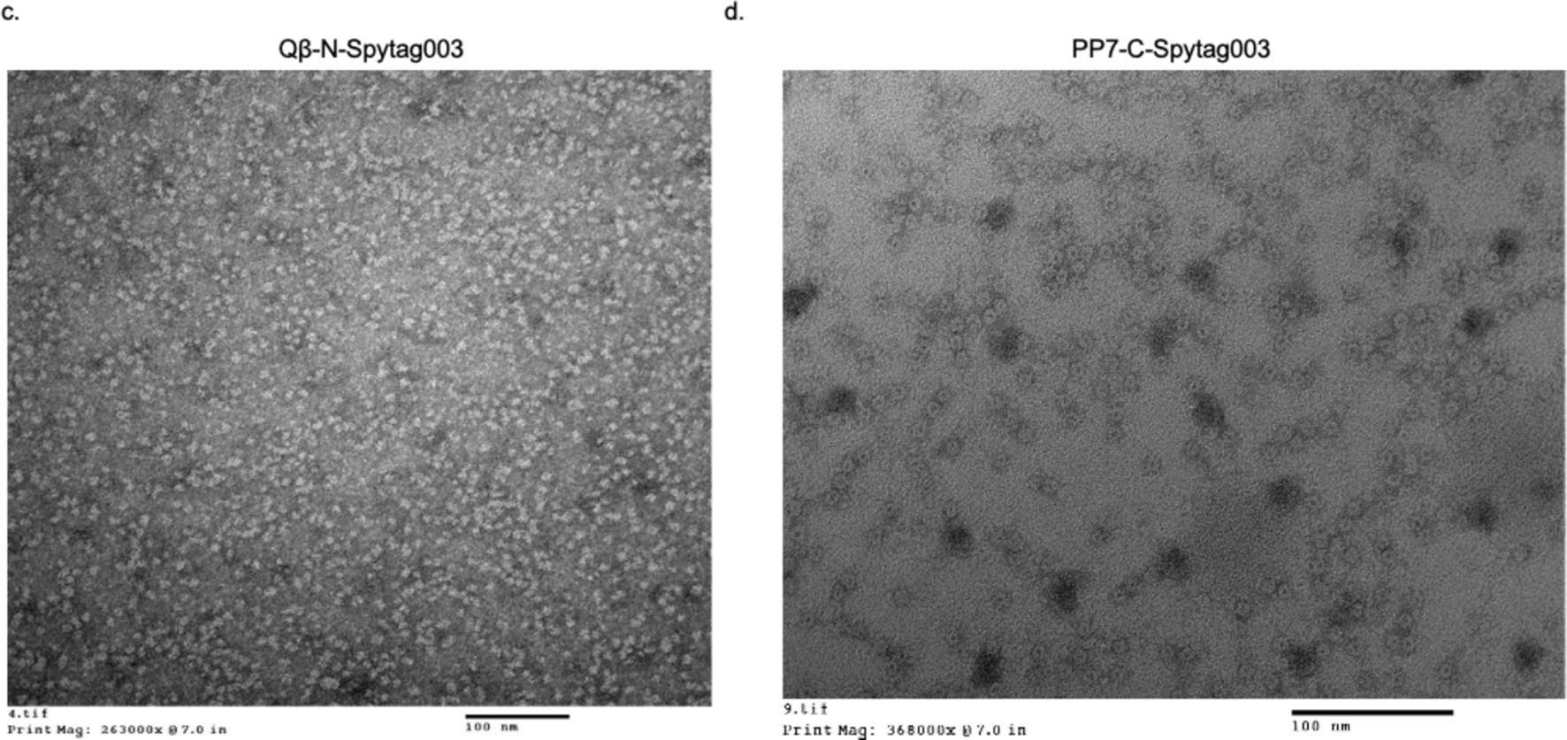
Purification of the coat proteins of Qβ-N-Spytag003 coat protein and PP7-C-Spytag003 coat protein. Bacterial pellet expressing **a** Qβ-N-Spytag003 coat protein and **b** PP7-C-Spytag003 coat protein were sequential lysed with SCB buffer, Borax, and BugBuster reagent followed by lysis with 0.5 M, 1 M, and 8 M urea. Supernatant after each lysis were run on SDS-PAGE gel. Qβ-N-Spytag003 coat proteins (lysed with 0.5 and 1 M urea) were combined and was dialyzed (buffer exchanged with PBS) using Amicon Ultra Centrifugal Filter, 3 KDa molecular weight cutoff. Samples were on SDS-PAGE [the right of panel (a)]. Positive controls represent the lysates of Qβ-N-Spytag003 coat protein and PP7-C-Spytag003 coat protein lysed with 8 M urea. **c** and **d** TEM images of negatively stained sample of concentrated Qβ-N-Spytag003 from panel (a) and PP7-C-Spytag003 samples from panel (b), respectively. TEM were taken at 50,000 X and 70,000X, respectively

Attempts to purify the samples by ultracentrifugation using 23, 29, and 35% Opti Prep density gradients or 29, 35, and 41% Opti Prep density gradients supplemented with 0.1% Triton-X 100 were unsuccessful (Supplemental Fig. 1). In most of the layers of samples collected after ultracentrifugation, Qβ-N-Spytag003 and PP7-C-Spytag003 coat proteins co-purified with contaminating bacterial proteins. Sequential lysis of bacterial pellet (expressing the recombinant coat proteins) with SCB, Borax, and BugBuster, followed by lysis with increasing concentrations of urea (0.5–8 M), removed some of the contaminating bacterial proteins and improved solubility of the proteins (Fig. 4A and B). To assess whether some of the solubilized proteins with low concentration of urea (0.5 and 1 M) were VLPs, we did buffer exchange (of urea) with PBS buffer and concentrated the samples & analyzed them under the TEM. Unfortunately, only small oval to irregular-shaped protein structures (particles), ~ 5 nm for Qβ-N-Spytag003 and ~ 11–14 nm for PP7-C-Spytag003, were observed under TEM (Fig. 4C and D).

### The Insertions of Spytag into the *N* Terminus or the AB-Loop of the Coat Protein of PP7 Did Not Affect the Ability of the Coat Protein to Assemble into VLPs

**Fig. 5:**
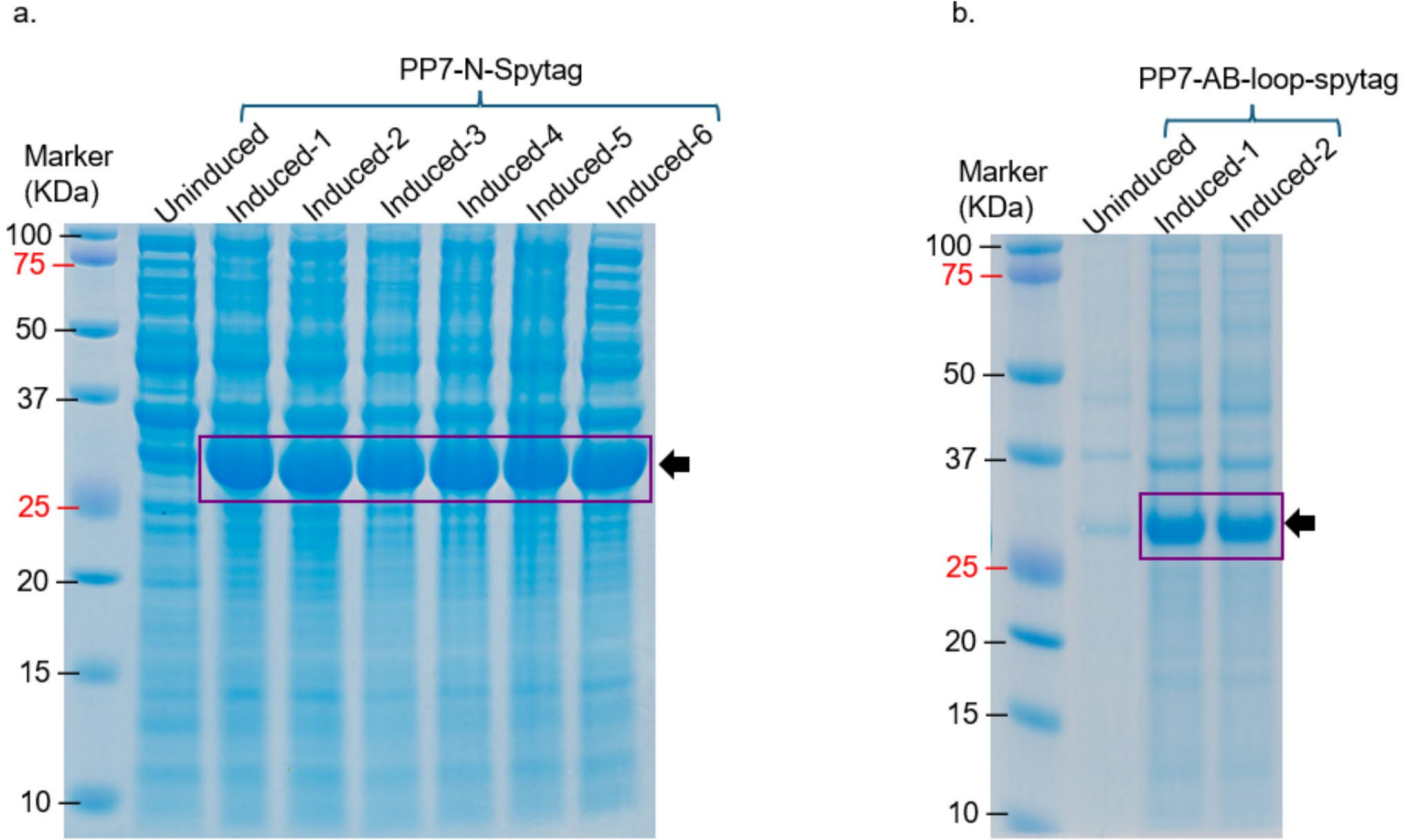
SDS-PAGE of small-scale expression of the recombinant coat proteins of PP7-Spytag. Expression of **a** PP7-N-Spytag and **b** PP7-AB-loop- Spytag in C41 cells. In all cases, recombinant coat protein was induced with 0.5-mM IPTG at 37 °C for 4 h. Four hours after induction, bacteria were pelleted, lysed with 8 M urea and lysates were run on SDS-PAGE gels. Lanes labeled 1–6 correspond to independent colonies that were picked after transformation of the plasmid into competent cells. Arrows indicate expected protein size ~ 30.59 KDa)

**Fig. 6:**
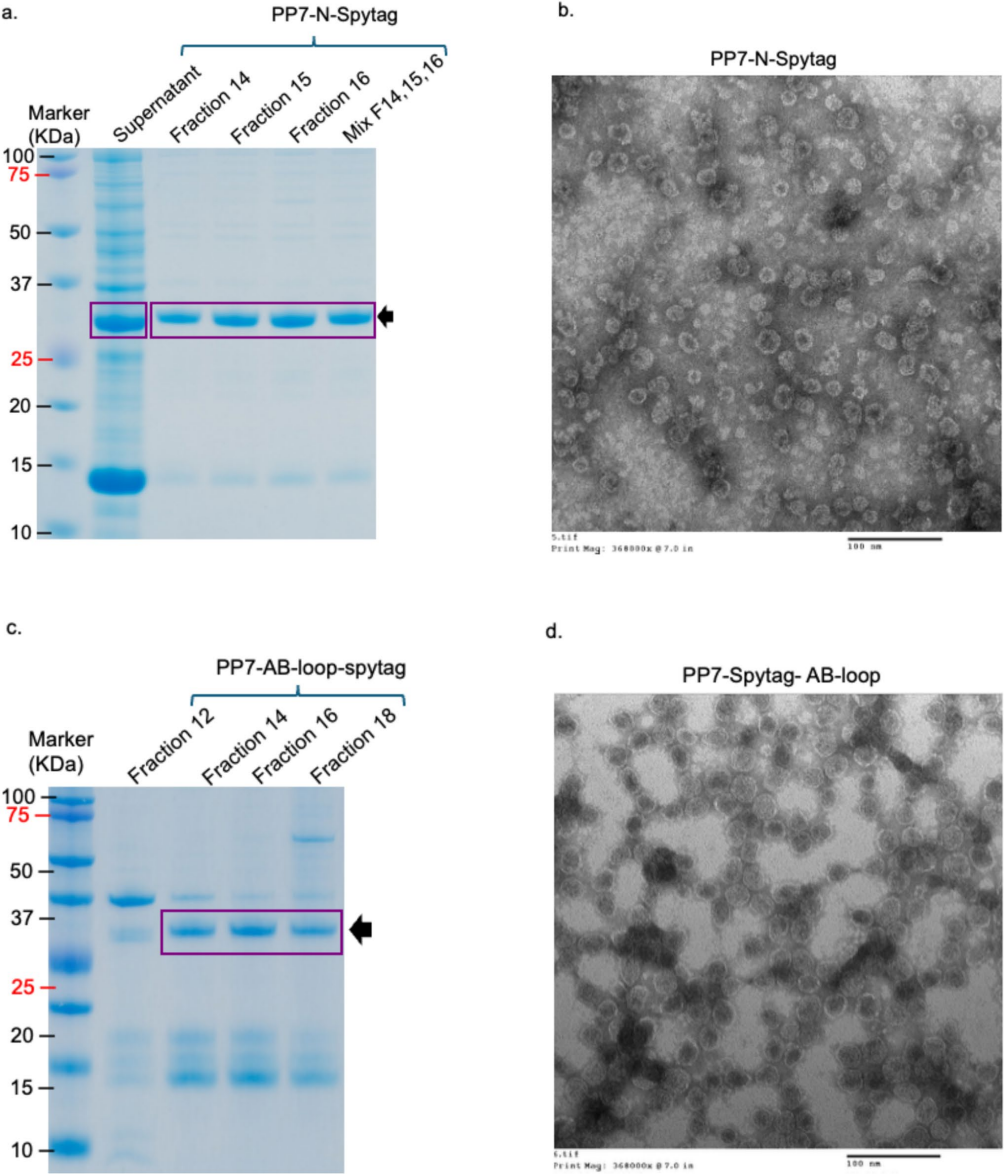
Purification and TEM analysis of PP7-N-Spytag and PP7-AB-loop-Spytag. **a** SDS-PAGE analysis of purified fractions (by SEC using a Sepharose CL-4B column) of expressed PP7-N-Spytag coat protein. **b** TEM image of purified PP7-N-Spytag VLPs. **c** SDS-PAGE analysis of purified fractions (by SEC using a Sepharose CL-4B column) of expressed PP7-AB-loop-Spytag coat protein. **d** TEM analysis of purified PP7-AB-loop-Spytag. Arrows indicate expected size (~ 30.59 KDa) of recombinant coat protein. TEM images were taken at 70,000X. Sizes of LVPs are ~ 27–30 nm

Given the fact that the insertion of Spytag003 on the coat proteins (PP7 and Qβ) above did not lead to assembly of the coat proteins into VLPs, we explored the insertion of Spytag into the *N* terminus or the AB-loop (between residues 138 and 139) of the coat protein of the single-chain dimer of PP7. As mentioned above, Spytag is three amino acids shorter than Spytag003 and differs from Spytag003 by 6 amino acids (Fig. 1). Similar to the insertion of Spytag003, the insertion of Spytag on the coat protein of PP7 (*N* terminus or AB-loop) did not affect the expression of the coat proteins (Fig. 5). However, unlike Spytag003 insertions which affected assembly to VLPs, the insertion of Spytag did not affect the assembly of the coat proteins into VLPs. As shown in Fig. 6, purified PP7 coat proteins (expected size of ~ 30.59 KDa) with Spytag insertion at either the *N* terminus or the AB-loop assembled into VLPs with diameters of 27–30 nm.

### Chimeric PP7-N-Spytag VLPs Could be Conjugated with Spycatcher003 Protein and SpyCatcher003-Ag2/PRA-CSA Protein

**Fig. 7:**
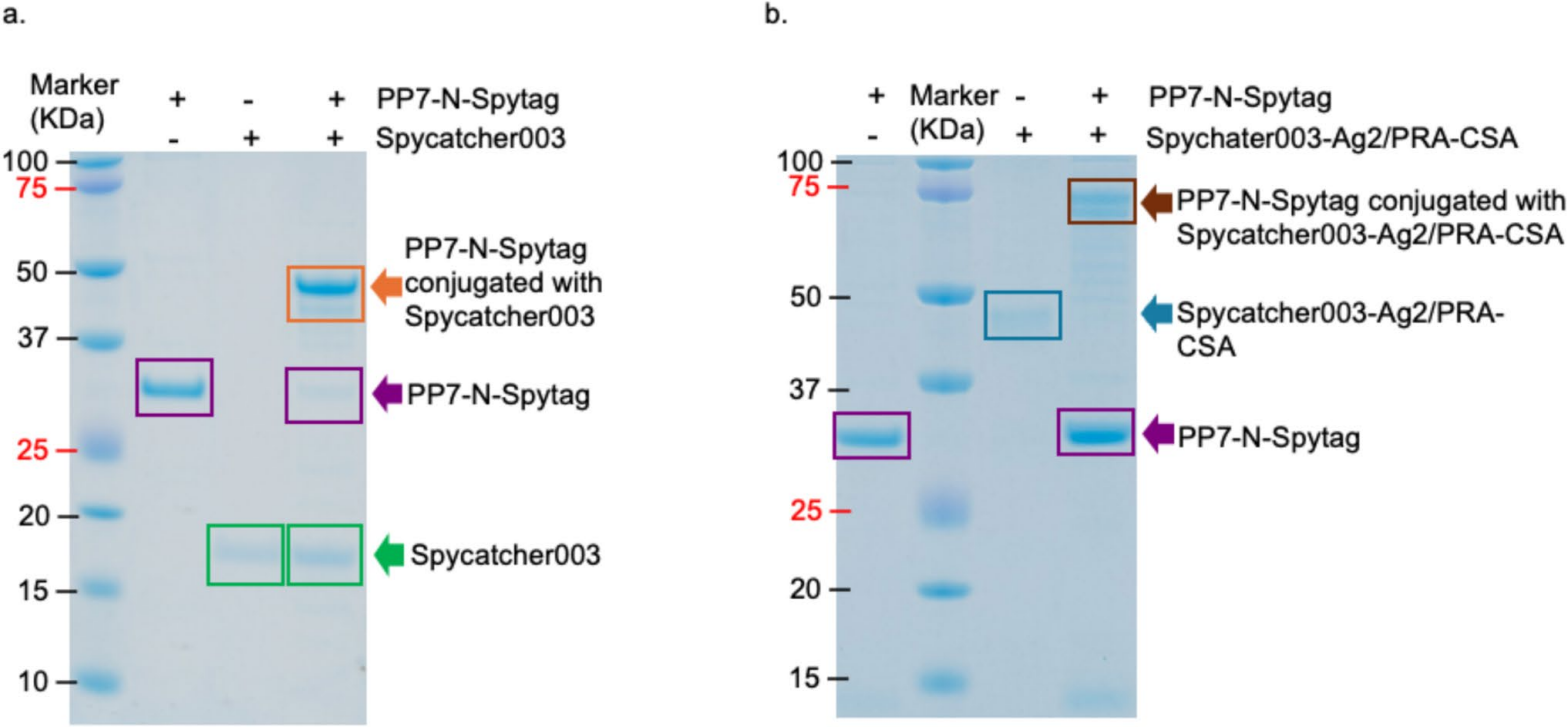
Conjugation of PP7-N-Spytag VLPs with Spycatcher003 and Spycatcher003-Ag2/PRA-CSA. **a** SDS-PAGE showing conjugation of PP7-N-Spytag with Spycatcher003 at a molar ratio of 1:3.25 (VLPs:Spycatcher003). PP7-N-Spytag alone (~ 30.59 KDa); Spycatcher003 alone (~ 15.15 KDa); conjugation reaction, showing the up-shifted PP7-Spytag-Spycatcher003 complex (~ 45.6 KDa). **b** SDS-PAGE showing conjugation of PP7-N-Spytag with Spycatcher003-Ag2/PRA-CSA at a molar ratio of 1:0.43 (VLPs:Spycatcher3-Ag2/PRA-CSA). Spycatcher3-Ag2/PRA-CSA alone (~ 43.5 kDa); conjugation reaction, showing the up-shifted PP7-Spytag-Spycatcher003-Ag2/PRA-CSA complex (~ 70–73 KDa). All conjugation reactions were done at room temperature for 3 h. Conjugation efficiency was calculated by subtracting unconjugated coat protein from total coat protein before conjugation, divided by the total coat protein before conjugation

To evaluate whether Spytag was displayed on the VLPs, we first explored the conjugation of Spytag binding partner-related protein (Spycatcher003) on PP7-N-Spytag VLPs. Spycatcher003 differs from the prototype Spytag binding partner (Spycatcher) by only 13 amino acids. The protein is available commercially; thus, we decided to use it to assess the display of Spytag on PP7 VLPs. The molar ratio of 1:3.25 (VLP:Spycatcher003) had the highest rate of conjugated protein with the conjugation efficiency of 84%. As shown in Fig. 7A, Spycatcher003 was successfully conjugated on PP7-N-Spytag VLPs. To assess whether a recombinant protein attached to Spycatcher003 could also be conjugated to the VLPs, we expressed and purified a recombinant fungal antigen (Ag2/PRA-CSA) linked to Spycatcher003. Spycatcher003-Ag2/PRA-CSA was also successfully conjugated on the PP7-N-Spytag VLPs; PP7-N-Spytag VLPs conjugated with Spycatcher003-Ag2/PRA-CSA migrated at ~ 70–73 KDa compared to unconjugated samples which migrated at lower molecular weights (~ 30.59 KDa for PP7-N-Spytag VLPs and ~ 43.5 KDa for Spycatcher003-Ag2/PRA-CSA; Fig. 7B). The conjugation of Spytag003-Ag2/PRA-CSA on PP7-AB-loop VLPs was not successful suggesting that the insertion in the AB-loop may not be freely accessible compared to insertion in the *N* terminus.

### Spycatcher003 Protein Conjugated on PP7-N-Spytag VLPs Elicited High Titer Antibodies Compared to Unconjugated Protein

**Fig. 8:**
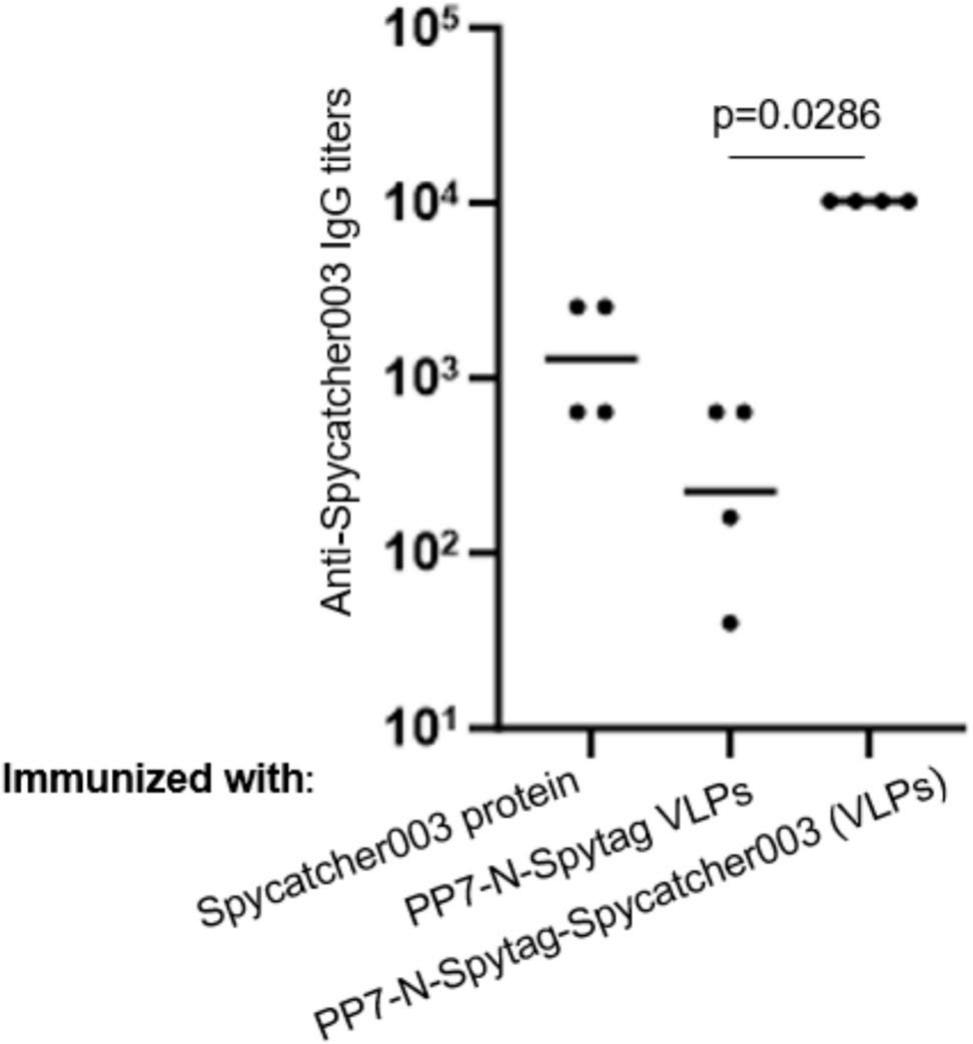
Serum anti-SpyCatcher003 IgG titers measured by endpoint dilution ELISA 2 weeks after the second immunization. Groups of Balb/c mice were immunized with Spycatcher003 protein alone, unconjugated PP7-N-Spytag VLPs, or PP7-N-Spytag VLPs conjugated with Spycatcher003 (PP7-N-Spytag–Spycatcher003 VLPs). Serial serum dilutions were tested, and endpoint titers were defined as the highest dilution yielding an optical density above background. Each point represents an individual mouse, with horizontal bars indicating group means. Mice immunized with PP7-N-Spytag–Spycatcher003 VLPs exhibited significantly higher endpoint IgG titers compared with Spycatcher003 protein (*p* = 0.026) and unconjugated PP7-N-Spytag VLPs (*p* = 0.029). Statistical comparisons were performed using the Mann–Whitney *U* test, with *p* < 0.05 considered statistically significant

To assess whether conjugated protein (Spycatcher003) can elicit better immune responses compared to unconjugated protein, we immunized Balb/c mice. Mice immunized with Spycatcher003 conjugated on PP7-N-Spytag VLPs elicited antibody titers that were significantly higher (*p* = 0.0286) than those immunized with the unconjugated protein (Fig. 8).

## Discussion

VLPs are increasingly being used as a display platform to enhance the immunogenicity of peptide and some protein antigens [7, 8]. More recently, the Spytag/Spycatcher system has been used to conjugate foreign antigens on the surface of VLPs notably VLPs derived from bacteriophage AP205, norovirus, hepatitis B, porcine circovirus type 2, etc. [13–18]. While the Spytag/Spycatcher conjugation system enables the conjugation of foreign antigens and the induction of high-titer antibodies against antigens of interest in pre-clinical models, this prototype conjugation system has high levels of pre-existing antibodies in the human population probably due to exposure to *Streptococcus pyogenes*, the bacterium from which the conjugation system was derived [19, 28]. Thus, an alternative conjugation system, for vaccine design, with low levels of pre-existing antibodies in the human population is required. Additionally, more VLP platforms (aside from AP205, norovirus, hepatitis B, porcine circovirus type 2) with this type of conjugation technology, for heterologous prime-boost immunization regimens or developing candidate vaccines against different infectious agents are required. In this study, we assessed whether the insertion of Spytag003 into the coat proteins of two bacteriophages, PP7 (single-chain dimer) and Qβ, will prevent the recombinant coat proteins from assembling into VLPs with the long-term goal of using the chimeric VLPs to display foreign antigens for vaccine studies. Spytag003 and its binding partner (Spycatcher003) have low levels of pre-existing antibodies in the public compared to the prototype system; additionally, the conjugation time between the former is shorter (less than 3 h) compared to the latter. Surprisingly, we observed that the coat proteins were expressed at different levels (or no visible expression at all) based on where Spytag003 was inserted. For example, insertion of Spytag003 at the *N* terminus of the coat protein of Qβ led to better expression compared to insertion of the peptide at the *C* terminus. Conversely, expression was not even visible when the peptide was inserted at the *N* terminus (compared to the *C* terminus) of the coat protein of PP7 single-chain dimer regardless of the expression system (Fig. 2). It is not clear to us why these insertions reduced or prevented expression of the coat proteins, especially at the *N* terminus of PP7. We do not believe RNA instability may have been the problem given the fact that attempts to express the recombinant proteins in a strain of *E. coli* (BL21 Star) that enhances RNA stability was futile. With that said, we noticed that insertion of the prototype Spytag, which is only 3 amino acids shorter and 6 amino acids different from Spytag003 had no effect in expression (Fig. 5A). We speculate that the insertion of Spytag003 peptide at the *N* terminus of PP7 may have led to the formation of RNA secondary structures, which may have prevented translation of the recombinant protein. Studies have shown that secondary structures (stem loops, especially those that are stable) near the start codon can negatively impact translation [29–33]. When we predicted the RNA secondary structures of PP7-N-Spytag003 and PP7-N-Spytag, and observed that PP7-N-Spytag003 has three stem loops around the start codon, while PP7-N-Spytag has only one stem loop (Supplemental Fig. 2); this may explain why PP7-N-Spytag003 was not expressed.

While the insertion of Spytag003 on the *N* terminus of Qβ and the *C* terminus of PP7 did not affect the ability of the recombinant coat proteins to be expressed, the insertions however affected the solubility of the coat proteins. Attempts to solubilize the proteins (and eliminate contaminating bacterial proteins) using sequential lysis and with low concentration of urea were successful to an extent. Unfortunately, TEM analysis of dialyzed proteins (by buffer exchange) only showed protein particles (Fig. 4C and D). It is not clear whether the expressed proteins formed VLPs and were denatured during lysis with 0.5–1 M urea. Although these protein particles look like monomeric forms of VLPs that have been disassembled [34], we suspect the expressed proteins did not assemble into VLPs given the fact that they were not soluble. In our experience, recombinant coat proteins that are soluble in solution after lysis are assembled VLPs.

Unlike the insertion of Spytag003, the insertion of prototype Spytag at the *N* terminus and the AB loop of the single-chain dimmer of PP7 was soluble and the recombinant coat protein formed VLPs (Fig. 6). Although we did not try to insert the prototype Spytag into the *N* terminus of Qβ (a limitation of this study) to assess if this peptide would affect assembly in comparison to Spytag003, which prevented assembly, we believe the size of the peptide, the amino acid composition, and the length of the linkers may have affected assembly into VLPs. As mentioned above, Qβ-N-Spytag003 was expressed but not soluble while PP7-N-Spytag was soluble. Although these are two different coat proteins, Spytag003 inserted at the *N* terminus of Qβ is three amino acids longer than Spytag that was inserted at the *N* terminus of PP7. In addition to this, the amino acid composition of Spytag003 differs from that of prototype Spytag by six amino acids; this difference in amino acids makes Spytag003 more hydrophobic compared to the prototype Spytag (hydrophobicity 32.85 vs 28.60, respectively). We speculate that the hydrophobic nature of Spytag003 may have affected the solubility of the recombinant coat proteins to which it was inserted and consequently, the inability of the proteins to assemble to VLPs. This view is supported by studies that have shown that the insertion of hydrophobic epitopes on coat proteins can affect the solubility of the recombinant coat protein as well as its potential to assemble to VLPs [35–37]. Another difference between Spytag003 insertions and prototype Spytag insertion is the size of the linkers that were used. Spytag003 insertions had 9-amino acids linker sequences (GSGGSGGSG) because the peptide is longer, requiring greater spatial separation from the VLP surface unlike Spytag insertions, which was made up of only 4 linkers (GGGS) consistent with prior successful PP7 insertion strategies [38]. Any of these differences in linker sizes (which ultimately increased the overall length of foreign peptides inserted) could have affected assembly. Thus, studies are required to assess the effect of linker-length on Spytag insertions on VLP assembly.

The coat protein of PP7 with Spytag insertion at the *N*-terminus or the AB-loop assembled to VLPs. Spytag was exposed on the surface of VLPs (especially PP7-N-Spytag) as confirmed by the conjugation of Spytag003 protein (~ 15.15 KDa) and a fungal antigen, Ag2/PRA-CSA, linked to Spytag003 (a ~ 43.5 KDa complex); Fig. 7. To our surprise, Spycatcher003 conjugated to Spytag on the VLPs even though the former differs from the prototype Spycatcher (the binding partner of Spytag) by 13 amino acids. As mentioned in the introduction, antibodies against prototype Spycatcher are high in population compared to Spycatcher003, which makes Spycatcher less ideal as a conjugation system for vaccine design. Given the fact that Spycatcher003 can bind covalently to the prototype Spytag—proof of concept—foreign proteins of interest (for vaccine studies) can be expressed as recombinant proteins linked to Spytag003 and conjugated to the PP7-N-Spytag VLPs. Although we did not assess the immunogenicity of Ag2/PRA-CSA conjugated on PP7-N-Spytag VLPs (due to limited resources), immunogenicity of Spycatcher003 protein conjugated on the VLPs was significantly higher than that of unconjugated Spycatcher003 protein based on the Mann–Whitney analysis. These immunizations were performed strictly as pilot proof-of-concept experiments to confirm that antigen display on PP7 VLPs (using the Spytag/Spycatcher system) enhances antibody responses compared to unconjugated antigen. It is worth mentioning that the immune responses were elicited (with low doses of antigens, 1.5 ug of Spycatcher003 protein used for conjugation and immunizations) in the absence of exogenous adjuvants; this further underscores the importance of multivalent display of antigens of VLPs.

## Conclusion

The development of more VLP platforms such as PP7-N-Spytag (developed here) has the potential to add more VLPs in the “vaccine toolbox” of VLPs (in addition to AP205, norovirus, hepatitis B) which can be used in prime boost regimens or to develop new candidate vaccines against other infectious agents by displaying different antigens on different VLP platforms to minimize any potentials for anti-platform immune responses.

## Data Availability

Additional data contained within this article are included in the Supplementary Materials.
